# Public Attitudes, Behaviors, and Beliefs Related to COVID-19, Stay-at-Home Orders, Nonessential Business Closures, and Public Health Guidance — United States, New York City, and Los Angeles, May 5–12, 2020

**DOI:** 10.15585/mmwr.mm6924e1

**Published:** 2020-06-19

**Authors:** Mark É. Czeisler, Michael A. Tynan, Mark E. Howard, Sally Honeycutt, Erika B. Fulmer, Daniel P. Kidder, Rebecca Robbins, Laura K. Barger, Elise R. Facer-Childs, Grant Baldwin, Shantha M.W. Rajaratnam, Charles A. Czeisler

**Affiliations:** ^1^Monash University, Melbourne, Australia; ^2^Austin Health, Melbourne, Australia; ^3^CDC COVID-19 Response Team; ^4^University of Melbourne, Melbourne, Australia; ^5^Brigham and Women’s Hospital, Boston, Massachusetts; ^6^Harvard Medical School, Boston, Massachusetts.

SARS-CoV-2, the virus that causes coronavirus disease 2019 (COVID-19), is thought to be transmitted mainly by person-to-person contact (*1*). Implementation of nationwide public health orders to limit person-to-person interaction and of guidance on personal protective practices can slow transmission (*2*,*3*). Such strategies can include stay-at-home orders, business closures, prohibitions against mass gatherings, use of cloth face coverings, and maintenance of a physical distance between persons (*2*,*3*). To assess and understand public attitudes, behaviors, and beliefs related to this guidance and COVID-19, representative panel surveys were conducted among adults aged ≥18 years in New York City (NYC) and Los Angeles, and broadly across the United States during May 5–12, 2020. Most respondents in the three cohorts supported stay-at-home orders and nonessential business closures* (United States, 79.5%; New York City, 86.7%; and Los Angeles, 81.5%), reported always or often wearing cloth face coverings in public areas (United States, 74.1%, New York City, 89.6%; and Los Angeles 89.8%), and believed that their state’s restrictions were the right balance or not restrictive enough (United States, 84.3%; New York City, 89.7%; and Los Angeles, 79.7%). Periodic assessments of public attitudes, behaviors, and beliefs can guide evidence-based public health decision-making and related prevention messaging about mitigation strategies needed as the COVID-19 pandemic evolves.

During May 5–12, 2020, a total of 4,042 adults aged ≥18 years in the United States were invited to complete a web-based survey administered by Qualtrics, LLC.^†^ Surveys were conducted among residents of NYC and Los Angeles to enable comparison of the two most populous cities in the United States with each other and with the nationwide cohort (*4*). The nationwide survey did not exclude respondents from NYC and Los Angeles, but no respondent was counted in more than one cohort. Invited participants were recruited using methods to create panels representative of the 2010 U.S. Census by age, gender, race, and ethnicity (*5*). Overall, 2,402 respondents completed surveys (response rate = 59.4%); of these, 2,221 (92.5%) (United States cohort = 1,676, NYC cohort = 286, and Los Angeles cohort = 259) passed quality screening procedures^§^ (*5*); sample sizes provided a margin of error at 95% confidence levels of 2.4%, 5.7%, and 5.9%, respectively.

Questions about the effects of the COVID-19 pandemic focused on public attitudes, behaviors, and beliefs regarding stay-at-home orders, nonessential business closures, and public health guidance. Chi-squared statistics (threshold of α = 0.05) were calculated to examine differences between the survey cohorts and to examine potential associations between reported characteristics (gender, age, race, ethnicity, employment status, essential worker status, rural-urban residence, knowing someone with COVID-19, and knowing someone who had died from COVID-19). Jupyter Notebook (version 6.0.0; Project Jupyter) was used to conduct statistical analyses.

Among respondents in the U.S. cohort (1,676), 16.8% knew someone who had positive test results for COVID-19, compared with 42.0% of respondents in NYC and 10.8% in Los Angeles (Table 1); 5.9% of respondents in the U.S. survey cohort knew someone who had died from COVID-19, compared with 23.1% in NYC and 7.3% in Los Angeles.

**TABLE 1:** Self-reported characteristics of invited participants and survey respondents — United States, New York City, and Los Angeles,* May 5–12, 2020

Characteristic	%^†^						
	United States		New York City		Los Angeles		
	Invited	Responded	Invited	Responded	Invited	Responded	
	(N = 3,010)	(N = 1,676)	(N = 507)	(N = 286)	(N = 525)	(N = 259)	
**Gender**							
Female	55.9	56.1	52.9	55.2	52.4	52.9	
Male	44.0	43.9	47.1	44.8	47.6	47.1	
Other	0.1	0.0	0.0	0.0	0.0	0.0	
**Age group (yrs)**							
18–24	11.4	3.9	11.2	4.2	11.0	5.8	
25–34	14.8	8.5	18.5	11.5	18.1	10.4	
35–44	17.6	15.0	15.6	14.0	17.5	12.4	
45–54	17.6	19.0	15.0	13.6	16.4	18.5	
55–64	18.0	23.4	19.3	26.9	17.1	22.0	
≥65	20.6	30.2	20.3	29.7	19.8	30.9	
**Race**							
White	78.4	84.7	72.6	82.5	74.3	80.7	
Black or African American	9.2	5.0	11.2	4.5	9.1	4.6	
Asian	5.7	6.2	6.1	7.3	5.7	7.3	
Multiple Race/other**^§^**	6.7	4.2	10.1	5.6	10.9	7.3	
**Ethnicity**							
Hispanic or Latino	8.8	5.9	13.6	8.0	17.1	10.8	
Not Hispanic or Latino	91.2	94.1	86.4	92.0	82.9	89.2	
**Rural-urban residence classification^¶^**							
Rural	15.3	15.5	0.8	1.4	0.8	0.4	
Urban	84.7	84.5	99.2	98.6	99.2	99.6	
Employment status**							
Employed**^††^**	62.9	49.6	71.2	58.7	68.6	52.5	
Essential	—	23.4	—	16.1	—	23.2	
Nonessential	—	26.2	—	42.7	—	29.3	
Retired	24.4	34.9	19.9	29.4	21.0	32.8	
Unemployed	12.8	15.5	8.9	11.9	10.5	14.7	
**Know someone with positive test results for COVID-19**	—	16.8	—	42.0	—	10.8
**Know someone who died from COVID-19**	—	5.9	—	23.1	—	7.3

**Abbreviation:** COVID-19 = coronavirus disease 2019.

* The U.S. survey group did not exclude respondents from New York City and Los Angeles.

^†^ Totals might not all sum to 100 because of rounding.

^§^ The multiple race/other category includes respondents who self-reported as a race with <2.5% of respondents in any cohort (e.g., American Indian or Alaska Native, Native Hawaiian or Pacific Islander, or more than one race).

^¶^ Rural-Urban classification was determined according to the Federal Office of Rural Health Policy definition of rurality. https://www.hrsa.gov/rural-health/about-us/definition/datafiles.html.

** Employment status as of December 2019.

**^††^** Essential versus nonessential status was not assessed in relation to employment status among invited participants. Totals for this category do not all sum to 100 because of rounding.

Broad support for recommended COVID-19 mitigation strategies was found nationwide (Table 2). Overall, 79.5% of respondents in the U.S. cohort supported government-issued stay-at-home orders and nonessential business closures, whereas 86.7% in NYC and 81.5% in Los Angeles supported these measures. Further, 67.3% of respondents in the United States, 76.6% in NYC, and 69.1% in Los Angeles agreed that nonessential workers should stay home. The majority of respondents in NYC and Los Angeles and broadly across the United States agreed with public health guidelines, including recommendations for maintaining 6 feet of distance between persons (>87% in each area) and limiting gatherings to fewer than 10 persons (>82% in each area). At the time of the survey, most also agreed that dining inside restaurants should not be allowed, with agreement higher in NYC (81.5%) than in Los Angeles (71.8%) and in the United States overall (66.6%).

**TABLE 2:** Attitudes, behaviors, and beliefs related to COVID-19, stay-at-home orders, nonessential business closures, and public health guidance — United States (U.S.),* New York City (NYC), and Los Angeles (LA), May 5–12, 2020

Attitudes, behaviors, and beliefs	U.S.	NYC	LA	p-value^†^	p-value^†^	p-value^†^
	(N = 1,676)	(N = 286)	(N = 259)	U.S. versus NYC	U.S. versus LA	NYC versus LA
**Attitudes, no. of respondents (%)**						
**Support stay-at-home order and nonessential business closures**						
Yes	1,332 (79.5)	248 (86.7)	211 (81.5)	<0.05**^§^**	0.5097	0.1187
No	344 (20.5)	38 (13.3)	48 (18.5)			
**Nonessential workers should stay home**						
Agree	1,128 (67.3)	219 (76.6)	179 (69.1)	<0.05**^§^**	0.6722	<0.05**^§^**
Neither agree nor disagree	283 (16.9)	41 (14.3)	38 (14.7)			
Disagree	265 (15.8)	26 (9.1)	42 (16.2)			
**Persons should always keep ≥6-ft of physical distance**						
Agree	1,470 (87.7)	262 (91.6)	234 (90.3)	0.1242	0.4707	0.6377
Neither agree nor disagree	127 (7.6)	17 (5.9)	15 (5.8)			
Disagree	79 (4.7)	7 (2.4)	10 (3.9)			
**Groups of 10 or more persons should not be allowed**						
Agree	1,381 (82.4)	247 (86.4)	226 (87.3)	0.1245	0.1374	0.8130
Neither agree nor disagree	156 (9.3)	25 (8.7)	19 (7.3)			
Disagree	139 (8.3)	14 (4.9)	14 (5.4)			
**Dining inside restaurants should not be allowed**						
Agree	1,117 (66.6)	233 (81.5)	186 (71.8)	<0.05**^§^**	0.1769	<0.05**^§^**
Neither agree nor disagree	244 (14.6)	28 (9.8)	36 (13.9)			
Disagree	315 (18.8)	25 (8.7)	37 (14.3)			
**Behaviors, no. of respondents (%)**						
**In self-isolation** ^¶^						
Yes	1,296 (77.3)	242 (84.6)	215 (83.0)	<0.05**^§^**	<0.05**^§^**	0.6954
No	380 (22.7)	44 (15.4)	44 (17.0)			
**Keep ≥6 ft apart from others**						
Always	975 (58.2)	191 (66.8)	172 (66.4)	0.0653	0.1576	0.8331
Often	357 (21.3)	54 (18.9)	42 (16.2)			
Sometimes	138 (8.2)	16 (5.6)	17 (6.6)			
Rarely	69 (4.1)	10 (3.5)	10 (3.9)			
Never	137 (8.2)	15 (5.2)	18 (6.9)			
**Avoid groups of 10 or more persons**						
Always	1,259 (75.1)	222 (77.6)	196 (75.7)	0.7621	0.9568	0.8975
Often	181 (10.8)	32 (11.2)	29 (11.2)			
Sometimes	59 (3.5)	9 (3.1)	7 (2.7)			
Rarely	39 (2.3)	5 (1.7)	5 (1.9)			
Never	138 (8.2)	18 (6.3)	22 (8.5)			
**Been to a public area in the previous week**						
Yes	1,533 (91.5)	260 (90.9)	235 (90.7)	0.8436	0.7851	0.9381
No	143 (8.5)	26 (9.1)	24 (9.3)			
Wear cloth face covering when in public**						
Always	925 (60.3)	208 (80.0)	183 (77.9)	<0.05^†^	<0.05**^§^**	0.7659
Often	212 (13.8)	25 (9.6)	28 (11.9)			
Sometimes	134 (8.7)	14 (5.4)	16 (6.8)			
Rarely	63 (4.1)	5 (1.9)	3 (1.3)			
Never	199 (13.0)	8 (3.1)	5 (2.1)			
**Beliefs, no. of respondents (%)**						
**Believe community mitigation strategies are**						
Not restrictive enough	302 (18.0)	49 (17.4)	42 (16.3)	0.0500	0.1699	<0.05**^§^**
The right balance	1,112 (66.3)	204 (72.3)	163 (63.4)			
Too restrictive	262 (15.6)	29 (10.3)	52 (20.2)			
**Would feel safe if community mitigation strategies were lifted nationwide at the time of survey**						
Yes	431 (25.7)	53 (18.5)	69 (26.6)	<0.05**^§^**	0.8102	0.0304
No	1,245 (74.3)	233 (81.5)	190 (73.4)			
No, but would like restrictions lifted and accept risks	287 (17.1)	36 (12.6)	33 (12.7)			

**Abbreviation:** COVID-19 = coronavirus disease 2019.

* The U.S. survey group did not exclude respondents from NYC and LA.

^†^ Calculated with Chi-squared test of independence.

^§^ P-value is statistically significant (p<0.05).

^¶^ For this survey, self-isolating means having no contact with others outside of the respondent’s household unless required for essential services.

** Of respondents who reported having been in a public area in the preceding week.

Widespread adherence to recommended COVID-19 mitigation strategies was reported in all three cohorts. Overall, 77.3% of adults nationwide reported self-isolating,^¶^ with 84.6% reporting this behavior in NYC and 83.0% in Los Angeles. Most respondents (79.5%) in the United States also reported the behavior of always or often keeping ≥6 feet apart from others, with higher percentages reporting this behavior in NYC (85.7%) and Los Angeles (82.6%). Always or often avoiding groups of 10 or more persons was reported by >85% of adults in the three cohorts. Approximately 90% of respondents reported having been in a public area during the preceding week; among those, 74.1% nationwide reported always or often wearing cloth face coverings when in public, with higher percentages reporting this behavior in NYC (89.6%) and Los Angeles (89.8%).

Overall, 84.3% of adults in the U.S. survey cohort believed their state’s COVID-19 community mitigation strategies were the right balance or not restrictive enough, compared with 89.7% in NYC and 79.7% in Los Angeles. As well, 74.3% of respondents in the United States reported they would not feel safe if these restrictions were lifted nationwide at the time the survey was conducted, compared with 81.5% in NYC and 73.4% in Los Angeles. In addition, among those who reported that they would not feel safe, some indicated that they would nonetheless want community mitigation strategies lifted and would accept associated risks (17.1%, 12.6%, and 12.7%, respectively).

Reported prevalence of self-isolation and feeling safe if community mitigation strategies were lifted differed significantly by age, employment status, and essential worker status among adults in the U.S. survey cohort (Table 3). The percentage of respondents who reported that they were in self-isolation was highest among persons aged 18–24 years (92.3%) and lowest among those aged 45–54 years (71.5%). The percentage who reported that they would feel safe if community mitigation strategies were lifted was approximately twice as high among persons aged 18–24 as it was among those aged ≥65 years (43.1% versus 19.2%). Respondents who reported that they were essential workers** accounted for 47.2% of employed respondents in the U.S. cohort and were significantly less likely than were nonessential workers to report self-isolating (63.1% versus 80.6%). Essential workers were also significantly more likely than were nonessential workers to report that they would feel safe if COVID-19 community mitigation strategies were lifted (37.7% versus 23.7%).

**TABLE 3:** Attitudes, behaviors, and beliefs related to COVID-19, stay-at-home orders, nonessential business closures, and public health guidance, by respondent characteristics* — United States, May 5–12, 2020

By gender, age group, and ethnicity, %										
Attitudes, behaviors and, beliefs	Gender		Age group (yrs)						Ethnicity	
	Male	Female	18–24	25–34	35–44	45–54	55–64	≥65	Hispanic	Non- Hispanic
**Attitudes**										
**Support stay-at-home orders and nonessential business closures**										
Yes	76.3	81.9	84.6	85.2	83.7	75.2	76.0	80.4	83.8	79.2
p-value^†^	0.0521		0.1803						1.0	
**Nonessential workers should stay home**										
Agree	64.9	69.2	55.4	76.8	72.2	62.7	62.0	70.8	72.7	67.0
Disagree	17.8	14.2	13.8	7.7	11.5	20.7	19.6	14.4	11.1	16.1
p-value^†^	0.9043		<0.05^§^						1.0	
**Persons should always keep ≥6-ft of physical distance**										
Agree	86.5	88.6	73.8	82.4	86.9	85.0	91.1	90.5	77.8	88.3
Disagree	4.8	4.7	4.6	5.6	2.8	7.2	4.8	3.8	6.1	4.6
p-value^†^	1.0		<0.05^§^						<0.05^§^	
**Groups of 10 or more persons should not be allowed**										
Agree	80.4	84.0	70.8	80.3	83.7	76.8	82.9	87.0	80.8	82.5
Disagree	9.9	7.0	10.8	8.5	6.0	11.9	9.2	6.1	5.1	8.5
p-value^†^	0.7238		<0.05^§^						1.0	
**Dining inside restaurants should not be allowed**										
Agree	62.2	70.1	67.7	72.5	68.3	60.8	65.6	68.6	66.7	66.6
Disagree	21.8	16.5	9.2	12.0	15.9	23.8	23.2	16.8	14.1	19.1
p-value^†^	<0.05^§^		<0.05^§^						1.0	
**Behaviors**										
**In self-isolation**										
Yes	75.8	78.5	92.3	81.7	77.8	71.5	72.7	81.2	87.9	76.7
p-value^†^	1.0		<0.05^§^						0.1246	
**Keep ≥6 ft apart from others**										
Always	54.6	61.0	29.2	56.3	60.3	55.2	56.4	64.6	54.5	58.4
Often	22.6	20.3	30.8	23.2	18.3	21.6	23.5	19.2	18.2	21.5
Sometimes	9.0	7.7	26.2	7.0	9.1	9.1	7.7	5.7	14.1	7.9
Rarely	5.0	3.4	9.2	5.6	2.8	4.4	4.6	3.2	7.1	3.9
Never	8.8	7.7	4.6	7.7	9.5	9.7	7.9	7.3	6.1	8.3
p-value^†^	0.7508		<0.05^§^						0.8299	
**Avoid groups of 10 or more persons**										
Always	72.5	77.2	52.3	68.3	74.2	73.4	73.7	82.6	63.6	75.8
Often	12.2	9.7	15.4	18.3	11.9	8.8	12.0	7.9	14.1	10.6
Sometimes	3.9	3.2	15.4	2.1	4.4	4.4	3.1	1.8	6.1	3.4
Rarely	2.4	2.2	15.4	2.8	0.4	2.2	2.0	1.8	6.1	2.1
Never	8.8	7.8	1.5	8.5	9.1	11.3	9.2	5.9	10.1	8.1
p-value^†^	1.0		<0.05^§^						0.1843	
**Been to a public area in the preceding week**										
Yes	94.7	88.9	96.9	88.0	92.5	90.6	94.4	89.5	90.9	91.5
p-value^†^	<0.05^§^		0.3145						1.0	
**Wear cloth face covering when in public^¶^**										
Always	54.6	65.1	44.4	59.2	57.9	56.1	55.1	71.1	57.8	60.5
Often	14.9	12.9	15.9	16.0	12.9	13.1	17.6	10.8	13.3	13.9
Sometimes	10.1	7.6	15.9	8.8	8.6	8.7	10.3	6.6	13.3	8.5
Rarely	4.6	3.7	12.7	4.0	4.7	4.5	3.5	2.9	4.4	4.1
Never	15.8	10.6	11.1	12.0	15.9	17.6	13.5	8.6	11.1	13.1
p-value^†^	<0.05^§^		<0.05^§^						1.0	
**Beliefs**										
**State restrictions are**										
The right balance	64.5	67.8	61.5	57.0	65.1	63.3	67.3	71.3	60.6	66.7
Not restrictive enough	18.0	18.1	21.5	31.7	19.0	16.9	16.1	15.4	26.3	17.5
p-value^†^	1.0		<0.05^§^						0.7720	
**Would feel safe if restrictions were lifted nationwide at the time the survey was conducted**										
Yes	28.8	23.3	43.1	26.8	27.4	30.1	26.3	19.2	25.3	25.7
p-value^†^	0.1019		<0.05^§^						1.0	
**By race, employment status, and essential worker status, %**										
**Attitudes, behaviors, and beliefs**	Race**				**Employment status**				**Essential worker^††^**	
	**White**	**Black**	**Asian**	**Multiple race/Other**	**Unemployed**		**Retired**	**Employed**	**Yes**	**No**
**Attitudes**										
**Support stay-at-home orders and nonessential business closures**										
Yes	77.9	89.2	90.4	84.3	81.9		80.0	78.4	75.6	80.9
p-value^†^	<0.05^§^				1.0				0.6953	
**Nonessential workers should stay home**										
Agree	66.4	63.9	78.8	72.9	68.3		69.9	65.1	58.3	71.3
Disagree	16.8	16.9	4.8	11.4	13.9		14.9	17.1	19.6	14.8
p-value^†^	0.4225				1.0				<0.05^§^	
**Persons should always keep ≥6-ft of physical distance**										
Agree	88.2	81.9	89.4	81.4	83.0		92.5	85.8	81.7	89.5
Disagree	4.9	6.0	1.9	4.3	8.1		2.1	5.5	7.1	4.1
p-value^†^	1.0				<0.05^§^				<0.05^§^	
**Groups of 10 or more persons should not be allowed**										
Agree	82.0	84.3	89.4	78.6	79.5		87.5	79.7	74.8	84.1
Disagree	8.9	7.2	1.9	7.1	9.7		5.8	9.6	10.7	8.7
p-value^†^	1.0				<0.05^§^				<0.05^§^	
**Dining inside restaurants should not be allowed**										
Agree	65.8	75.9	72.1	64.3	66.0		69.6	64.8	59.5	69.5
Disagree	20.5	7.2	6.7	15.7	19.3		16.9	20.0	22.4	17.8
p-value^†^	<0.05^§^				1.0				0.0899	
**Behaviors**										
**In self-isolation**										
Yes	77.2	78.3	73.1	84.3	81.1		82.7	72.4	63.1	80.6
p-value^†^	1.0				<0.05^§^				<0.05^§^	
**Keep ≥6 ft apart from others**										
Always	58.2	48.2	67.3	55.7	58.3		65.8	52.8	44.8	59.9
Often	21.6	20.5	17.3	21.4	21.6		19.0	22.8	26.0	20.0
Sometimes	8.0	14.5	4.8	11.4	5.8		5.5	10.9	13.0	9.1
Rarely	3.9	9.6	1.0	5.7	5.4		2.9	4.6	6.6	2.7
Never	8.2	7.2	9.6	5.7	8.9		6.8	8.9	9.7	8.2
p-value^†^	0.5507				<0.05^§^				<0.05^§^	
**Avoid groups of 10 or more persons**										
Always	76.2	56.6	77.9	71.4	73.0		81.2	71.5	65.6	76.8
Often	10.8	15.7	6.7	11.4	10.8		8.2	12.6	16.0	9.6
Sometimes	3.0	12.0	1.9	5.7	4.2		2.2	4.2	5.6	3.0
Rarely	2.0	8.4	1.9	2.9	2.3		2.1	2.5	4.1	1.1
Never	8.0	7.2	11.5	8.6	9.7		6.3	9.1	8.7	9.6
p-value^†^	<0.05^§^				0.1179				<0.05^§^	
**Been to a public area in the preceding week**										
Yes	91.8	91.6	87.5	91.4	88.4		89.1	94.1	97.5	91.1
p-value^†^	1.0				<0.05^§^				<0.05^§^	
**Wear cloth face covering when in public^¶^**										
Always	60.1	55.3	71.4	54.7	58.5		70.4	54.2	49.3	58.8
Often	13.7	19.7	9.9	14.1	10.0		11.1	16.7	20.4	13.3
Sometimes	8.4	13.2	8.8	10.9	10.5		5.6	10.3	9.7	11.0
Rarely	3.8	7.9	3.3	7.8	2.2		3.1	5.4	6.5	4.3
Never	14.0	3.9	6.6	12.5	18.8		9.8	13.4	14.1	12.8
p-value^†^	0.3708				<0.05^§^				0.1843	
**Beliefs**										
**State restrictions are**										
The right balance	66.7	65.1	67.3	60.0	67.6		68.7	64.3	64.9	63.8
Not restrictive enough	16.7	28.9	22.1	25.7	18.5		17.4	18.3	14.5	21.6
p-value^†^	0.0523				1.0				0.0563	
**Would feel safe if restrictions were lifted nationwide at the time the survey was conducted**										
Yes	25.8	37.3	15.4	25.7	22.4		20.7	30.3	37.7	23.7
p-value^†^	0.0765				<0.05^§^				<0.05^§^	

* Nationwide cohort (n = 1,676) only unless otherwise specified. The six respondent characteristic categories shown in the table (gender, age, ethnicity, race, employment status, and essential worker status) account for 32 of 34 significant associations among the 108 potential interactions evaluated. Responses and p-values values for significant associations with characteristics not presented in the table that are associated with the attitudes, behaviors, and beliefs include the following: Use of cloth face coverings when in public × Rural-urban classification, (p-value = 0.0324); Rural: Always = 51.4%, Often = 15.5%, Sometimes = 10.2%, Rarely = 7.8%, Never = 15.1%; Urban: Always = 62.0%, Often = 13.5%, Sometimes = 8.5%, Rarely = 3.4%, Never = 12.6%; attitude that dining inside restaurants should not be allowed × Know someone with COVID-19 (p-value = 0.0243), Know someone: Agree = 75.1%, Disagree = 12.5%; Do not know someone: Agree = 64.9%, Disagree = 20.1%.

^†^ Calculated with Chi-squared test of independence.

^§^ P-value is statistically significant.

^¶^ Of respondents who reported having been in a public area in the preceding week.

** The multiple race/other category includes respondents who self-reported as a race with <2.5% of respondents in any cohort (e.g., American Indian or Alaska Native, Native Hawaiian or Pacific Islander, or more than one race).

^††^ Of 832 employed respondents in the U.S. cohort.

Reported prevalences of always or often wearing a cloth face covering in public and maintaining ≥6 feet of physical distance also varied significantly across respondent demographics and characteristics. Respondents who were male, employed, or essential workers were significantly more likely to report having been in public areas in the past week. Among respondents who had been in public areas during the preceding week, significantly higher percentages of women, adults aged ≥65 years, retired persons, and those living in urban areas reported wearing cloth face coverings. A significantly higher percentage of adults aged ≥65 years and nonessential workers reported maintaining 6 feet of physical distance between themselves and others and abiding by the recommendation to avoid gatherings of 10 or more persons than did others. Adherence to recommendations to maintain 6 feet of physical distance and limit gatherings to fewer than 10 persons also differed significantly by employment status and race, respectively, with employed persons less likely than were retired persons to have maintained 6 feet of distance and black persons less likely than were white or Asian persons to have limited gatherings to fewer than 10 persons.

## Discussion

There was broad support for stay-at-home orders, nonessential business closures, and adherence to public health recommendations to mitigate the spread of COVID-19 in early- to mid-May 2020. Most adults reported they would not feel safe if government-ordered community mitigation strategies such as stay-at-home orders and nonessential business closures were lifted nationwide at the time the survey was conducted, although a minority of these adults who did not feel safe wanted these restrictions lifted despite the risks.

There was a significant association between age and feeling safe without community mitigation strategies, with younger adults feeling safer than those aged ≥65 years, which might relate to perceived risk for infection and severe disease. As of May 16, adults aged ≥65 years accounted for approximately 80% of reported COVID-19–associated deaths, compared with those aged 15–24 years, who accounted for 0.1% of such deaths (*6*). Identifying variations in public attitudes, behaviors, and beliefs by respondent characteristics can inform tailored messaging and targeted nonpharmacological interventions that might help to reduce the spread of COVID-19.

Other variations in attitudes, behaviors, and beliefs by respondent characteristics have implications for implementation of COVID-19 mitigation strategies and related prevention messaging. For example, a lower percentage of respondents in the U.S. survey cohort reported wearing cloth face coverings and self-isolating than did those in NYC and Los Angeles. However, although use of cloth face coverings in NYC and Los Angeles were similar, NYC experienced substantially higher COVID-19-related mortality during the initial months of the pandemic than did Los Angeles (*4*). Nationwide, higher percentages of respondents from urban areas reported use of cloth face coverings than did rural area respondents. Because outbreaks have been reported in rural communities and among certain populations since March 2020 (*7*,*8*), these data suggest a need for additional and culturally effective messaging around the benefits of cloth face coverings targeting these areas. Essential workers also reported lower adherence to recommendations for self-isolation, 6 feet of physical distancing, and limiting gatherings to fewer than 10 persons. These behaviors might be related to job requirements and other factors that could limit the ability to effectively adhere to these recommendations. Nevertheless, the high rate of person-to-person contact associated with these behaviors increases the risk for widespread transmission of SARS-CoV-2 and underscores the potential value of tailored and targeted public health interventions.

The findings in this report are subject to at least four limitations. First, behaviors and adherence to recommendations were self-reported; therefore, responses might be subject to recall, response, and social desirability biases. Second, responses were cross-sectional, precluding inferences about causality. Third, respondents were not necessarily representative among all groups; notably a lower percentage of African Americans responded than is representative of the U.S. population. In addition, participation might have been higher among persons who knew someone who had tested positive or had died from COVID-19, which could have affected support for and adherence to mitigation efforts. Finally, given that the web-based survey does not recruit participants using population-based probability sampling and respondents might not be fully representative of the U.S. population, findings might have limited generalizability. However, this survey did apply screening procedures to address issues related to web-based panel quality.

Widespread support for community mitigation strategies and commitment to COVID-19 public health recommendations indicate that protecting health and controlling disease are public priorities amid this pandemic, despite daily-life disruption and adverse economic impacts (*5*,*9*). These findings of high public support might inform reopening policies and the timelines and restriction levels of these mitigation strategies as understanding of public support for and adherence to these policies evolves. Absent a vaccine, controlling COVID-19 depends on community mitigation strategies that require public support to be effective. As the pandemic progresses and mitigation strategies evolve, understanding public attitudes, behaviors, and beliefs is critical. Adherence to recommendations to wear cloth face coverings and physical distancing guidelines are of public health importance. Strong public support for these behaviors suggests an opportunity to normalize safe practices and promote continued use of these and other recommended personal protective behaviors to minimize further spread of COVID-19 as jurisdictions reopen. These findings and periodic assessments of public attitudes, behaviors, and beliefs can also inform future planning if subsequent outbreak waves occur, and if additional periods of expanded mitigation efforts are necessary to prevent the spread of COVID-19 and save lives.

**Summary**

**What is already known about this topic?**

Stay-at-home orders and recommended personal protective practices were disseminated to mitigate the spread of COVID-19 in the United States.

**What is added by this report?**

During May 5–12, 2020, a survey among adults in New York City and Los Angeles and broadly across the United States found widespread support of stay-at-home orders and nonessential business closures and high degree of adherence to COVID-19 mitigation guidelines. Most respondents reported that they would feel unsafe if restrictions were lifted at the time of the survey.

**What are the implications for public health practice?**

Routine assessment of public priorities can guide public health decisions requiring collective action. Current levels of public support for restrictions and adherence to mitigation strategies can inform decisions about reopening and balancing duration and intensity of restrictions.

## Footnotes

* Respondents were informed that, for the survey, stay-at-home orders mean that all nonessential services (e.g., dine-in restaurants, bars, social venues, gyms, fitness studios, and convention centers) are shut down. Essential services (e.g., groceries, pharmacies, gas stations, food banks, convenience stores, and delivery restaurants) remain open. Banks, local governments, and law enforcement agencies also remain open. Persons are still allowed to leave their homes but encouraged to observe social distancing guidelines. Public events and gatherings are not allowed.

† Eligibility for the nationwide U.S. cohort was determined on the basis of informed consent, age, and residence within the United States. Therefore, consented adult potential respondents residing in NYC and Los Angeles metro areas were eligible to complete surveys as part of the nationwide U.S. or NYC and Los Angeles cohorts.

§ Qualtrics LLC data quality screening procedures included algorithmic and keystroke analysis for attention patterns, click-through behavior, duplicate responses, machine responses, and inattentiveness. Country-specific geolocation verification via IP address mapping was used to ensure respondents were from the United States. Respondents who failed an attention or speed check, along with any responses identified by the data scrubbing algorithms, were excluded from analysis.

¶ For this survey, self-isolating means having no contact with others outside of the respondent’s household unless required for essential services.

** The definition of essential workers was largely determined on a state-by-state basis.
